# Tissue oxygen saturation predicts response to breast cancer neoadjuvant chemotherapy within 10 days of treatment

**DOI:** 10.1117/1.JBO.24.2.021202

**Published:** 2018-10-19

**Authors:** Jeffrey M. Cochran, David R. Busch, Anaïs Leproux, Zheng Zhang, Thomas D. O’Sullivan, Albert E. Cerussi, Philip M. Carpenter, Rita S. Mehta, Darren Roblyer, Wei Yang, Keith D. Paulsen, Brian Pogue, Shudong Jiang, Peter A. Kaufman, So Hyun Chung, Mitchell Schnall, Bradley S. Snyder, Nola Hylton, Stefan A. Carp, Steven J. Isakoff, David Mankoff, Bruce J. Tromberg, Arjun G. Yodh

**Affiliations:** a University of Pennsylvania, Department of Physics and Astronomy, Philadelphia, Pennsylvania, United States; b University of Texas Southwestern, Department of Anesthesiology and Pain Management, Dallas, Texas, United States; c University of California, Beckman Laser Institute and Medical Clinic, Irvine, California, United States; d Brown University School of Public Health, Department of Biostatistics and Center for Statistical Sciences, Providence, Rhode Island, United States; e University of Southern California, Keck School of Medicine, Department of Pathology, Los Angeles, California, United States; f University of California Irvine, Department of Medicine, Irvine, California, United States; g Boston University, Department of Biomedical Engineering, Boston, Massachusetts, United States; h University of Texas MD Anderson Cancer Center, Department of Diagnostic Radiology, Houston, Texas, United States; i Thayer School of Engineering, Dartmouth College, Hanover, New Hampshire, United States; j Dartmouth-Hitchcock Medical Center, Department of Hematology and Oncology, Lebanon, New Hampshire, United States; k University of Pennsylvania, Department of Radiology, Philadelphia, Pennsylvania, United States; l Brown University School of Public Health, Center for Statistical Sciences, Providence, Rhode Island, United States; m University of California, Department of Radiology, San Francisco, California, United States; n Massachusetts General Hospital, Athinoula A. Martinos Center for Biomedical Imaging, Department of Radiology, Boston, Massachusetts, United States; o Massachusetts General Hospital, Department of Hematology and Oncology, Boston, Massachusetts, United States; p University of Pennsylvania, Division of Nuclear Medicine, Department of Radiology, Philadelphia, Pennsylvania, United States

**Keywords:** biomedical optics, neoadjuvant chemotherapy, diffuse optical spectroscopy, breast cancer, therapy monitoring, translational imaging

## Abstract

Ideally, neoadjuvant chemotherapy (NAC) assessment should predict pathologic complete response (pCR), a surrogate clinical endpoint for 5-year survival, as early as possible during typical 3- to 6-month breast cancer treatments. We introduce and demonstrate an approach for predicting pCR within 10 days of initiating NAC. The method uses a bedside diffuse optical spectroscopic imaging (DOSI) technology and logistic regression modeling. Tumor and normal tissue physiological properties were measured longitudinally throughout the course of NAC in 33 patients enrolled in the American College of Radiology Imaging Network multicenter breast cancer DOSI trial (ACRIN-6691). An image analysis scheme, employing z-score normalization to healthy tissue, produced models with robust predictions. Notably, logistic regression based on z-score normalization using only tissue oxygen saturation ($S_{t}O_{2}$) measured within 10 days of the initial therapy dose was found to be a significant predictor of pCR (AUC=0.92; 95% CI: 0.82 to 1). This observation suggests that patients who show rapid convergence of tumor tissue $S_{t}O_{2}$ to surrounding tissue $S_{t}O_{2}$ are more likely to achieve pCR. This early predictor of pCR occurs prior to reductions in tumor size and could enable dynamic feedback for optimization of chemotherapy strategies in breast cancer.

## 1 Introduction

Neoadjuvant chemotherapy (NAC) is a widely used treatment method for breast cancer that permits increased conservation of breast tissue during tumor resection and limits the need for axillary node treatment and surgery.^1^ In addition, pathologic complete response (pCR) to NAC, defined as no residual invasive carcinoma, has been correlated with improved survival compared to incomplete response.^2^^,^^3^ Unfortunately, this assessment occurs after the completion of NAC. The ability to predict response to NAC at an earlier timepoint during chemotherapy, by contrast, could enable physicians to dynamically optimize the treatment regimen, thereby avoiding unnecessary therapy doses, reducing tissue damage, and improving patient outcomes.

NAC response is typically evaluated with physical exams and radiologic imaging in current clinical practice. Unfortunately, these methods are inadequate predictors of pCR.^4^^–^^6^ Magnetic resonance imaging (MRI) provides better correlation with pathology than mammography or ultrasound.^7^ Broadly, functional monitoring techniques offer significantly improved correlation with response relative to structural imaging modalities. Magnetic resonance spectroscopy (MRS),^8^ contrast-enhanced MRI,^9^ and positron emission tomography (PET),^10^^–^^12^ have predictive value with respect to pCR, but MRI, MRS, and PET all have practical constraints, which limit the frequency of monitoring in clinical care. These limitations include cost, the use of contrast agents, and ionizing radiation for PET.

The present contribution investigates the utility of diffuse optical monitoring for prediction of pCR during NAC and adds an analysis to prior reports of a multicenter trial.^13^ Briefly, diffuse optical techniques measure functional hemodynamic properties of tissue with nonionizing near-infrared radiation. These optical methods are relatively low cost and can be employed at the bedside. Furthermore, the technology offers a quantitative tool to predict treatment outcome based on longitudinal measurements during therapy.^14^^,^^15^ Diffuse optical spectroscopic imaging (DOSI) and tomography (DOT) probe deeply, i.e., several centimeters, into tissue and provide information about tissue optical absorption ($\mu_{a}$) and reduced optical scattering ($\mu_{s}^{\prime }$), from which deoxygenated-(HHb) and oxygenated-hemoglobin (${\mathrm{HbO}}_{2}$) concentrations, as well as lipid and water ($H_{2}O$) concentrations can be deduced.^16^^,^^17^ The concentrations of HHb and ${\mathrm{HbO}}_{2}$, in turn, are readily utilized to calculate total hemoglobin concentration (${\mathrm{Hb}}_{T}$) and tissue oxygen saturation ($S_{t}O_{2}$). These parameters have been shown to discriminate malignant from healthy tissue in the breast,^18^^–^^23^ and several studies have employed DOSI techniques to explore functional changes in malignancies during NAC and have correlated these changes with response to therapy.^13^^,^^24^^–^^35^

We recently reported the first results of ACRIN-6691, an American College of Radiology Imaging Network (ACRIN) multicenter clinical trial of patients monitored longitudinally by DOSI throughout their NAC regimen.^13^ The primary aim of ACRIN-6691 was to evaluate whether a change in a particular DOSI endpoint, the tissue optical index (TOI), could be used to predict a clinical endpoint, pCR, by the midpoint of NAC, ∼2 to 3 months after the first infusion. The TOI combines tissue deoxyhemoglobin concentration (HHb), water, and lipid into a single index (see Sec. 2). In that initial study, we reported significant reductions in tumor to normal (T/N) TOI ratios for pCR subjects. A 40% or greater change in this parameter at midpoint, combined with baseline tumor $S_{t}O_{2}$ greater than median values (77%) was shown to be a promising predictor of pCR (AUC=0.83; 95% CI: 0.63 to 1).^13^

In this study, we explore the ACRIN-6691 secondary aim of predicting pCR much earlier in the 3- to 6-month NAC cycle by examining DOSI response parameters within 10 days of therapy initiation. To address this goal, we develop and retrospectively apply z-score normalization^21^ and a logistic regression algorithm^36^ to correlate DOSI-measured parameters of malignant breast lesions to subjects’ posttherapy pathologic response status. Our hypothesis is that identification and optimization of this z-score DOSI index could predict pCR to NAC at an early timepoint in the course of therapy, providing significant potential for clinical utility.

## 2 Materials and Methods

### 2.1 Trial Design and Subjects

Data for this study were collected during the ACRIN 6691 multisite trial using a DOSI instrument developed at the University of California, Irvine.^13^ Subjects provided written informed consent, and the HIPAA-compliant protocol and informed consent were approved by the American College of Radiology Institutional Review Board, the NCI Cancer Therapy Evaluation Program, and each site’s Institutional Review Board. All 60 enrolled subjects were females between the ages of 28 and 67 with biopsy-confirmed invasive ductal carcinomas and/or invasive lobular carcinomas of at least 2 cm in length along the greatest dimension. For each subject, the chemotherapy regimen was determined by the subject’s physician. Chemotherapy type was not controlled in this study, except that regimens were required to include at least one cytotoxic chemotherapeutic agent.

pCR to therapy was defined as no residual invasive primary carcinoma without regard to residual lymph node disease and was determined for each subject from postsurgery pathology reports. Subjects that achieved partial response were not distinguished from nonresponders because of statistical considerations, i.e., sample size, and due to the previously reported correlation between complete response and improved survival.^2^^,^^3^ Table 1 contains demographic information, as well as tumor histology and immunohistochemistry for complete and noncomplete responders.

**Table 1:** Subject and tumor characteristics. Demographic, histological, and immunohistochemical data are provided for all subjects and divided into complete responder (pCR) and noncomplete responder (non-pCR) groups. For histological information, IDC refers to invasive ductal carcinoma, ILC refers to invasive lobular carcinoma, and DCIS is ductal carcinoma in-situ. ER, PR, and Her2 represent estrogen receptor, progesterone receptor, and human epidermal growth factor receptor status, respectively.

	pCR (N=15)	Non-pCR (N=18)
Age (years)		
Mean±SD (range)	49.0±11.6 (30 to 67)	49.4±10.9 (28 to 66)
Menopausal status, n (%)		
Pre	5 (33)	9 (50)
Peri	1 (7)	2 (11)
Post	9 (60)	7 (39)
Maximum tumor size (mm)		
Mean±SD (range)	37.9±22.8 (12 to 95)	37.5±18.1 (11 to 75)
Histological findings, n (%)		
IDC	9 (60)	12 (67)
ILC	0 (0)	1 (6)
IDC/DCIS	4 (27)	5 (28)
IDC/ILC	1 (7)	0 (0)
Unknown	1 (7)	0 (0)
ER status, n (%)		
Positive	5 (33)	16 (89)
Negative	8 (53)	2 (11)
Unknown	2 (13)	0 (0)
PR status, n (%)		
Positive	5 (33)	11 (61)
Negative	8 (53)	7 (39)
Unknown	2 (13)	0 (0)
Her2 status, n (%)		
1	5 (33)	4 (22)
2	1 (7)	9 (50)
3	5 (33)	1 (6)
Unknown	4 (27)	4 (22)
Molecular subtype, n (%)		
Her2 positive	3 (20)	1 (6)
HR positive	0 (0)	2 (11)
Luminal A	0 (0)	3 (17)
Luminal B	6 (40)	11 (61)
Triple negative	4 (27)	1 (6)
Unknown	2 (13)	0 (0)

A number of enrolled subjects were excluded from the final data set. Of these, three subjects withdrew from the study. An additional 13 subjects were not included in the imaging analysis because of the following DOSI scan issues: mandatory baseline DOSI was not performed (n=1), baseline DOSI was nonevaluable (n=8), mandatory midtherapy DOSI was not performed (n=3), or too few normal region points were available (n=1). A DOSI scan was considered nonevaluable in case of unrealistic physiological values or incorrect instrument configuration. This decision was made on blinded, deidentified data using instrument calibration and raw data QC reports.^13^ A flowchart for this exclusion process can be found in Fig. 6 in the Appendix.

### 2.2 Optical Imaging Methods

The DOSI instrument used in this study combines multispectral frequency-domain and broadband diffuse optical spectroscopy to measure tissue concentrations of oxygenated hemoglobin (${\mathrm{HbO}}_{2}$), deoxygenated hemoglobin (HHb), water ($H_{2}O$), and lipid, as well as the tissue scattering amplitude (A) and power (b), as defined by a simplified Mie scattering model.^37^ The combination of these measured parameters permits calculation of total tissue hemoglobin concentration (${\mathrm{Hb}}_{T}={\mathrm{HbO}}_{2}+\mathrm{HHb}$), tissue oxygen saturation ($S_{t}O_{2}={\mathrm{HbO}}_{2}/{\mathrm{Hb}}_{T}$), and the tissue reduced scattering coefficient ($\mu_{s}^{\prime }$). For a full description of the DOSI method and instrument performance in the multicenter ACRIN-6691 trial, see Ref. 38.

**Fig. 1:**
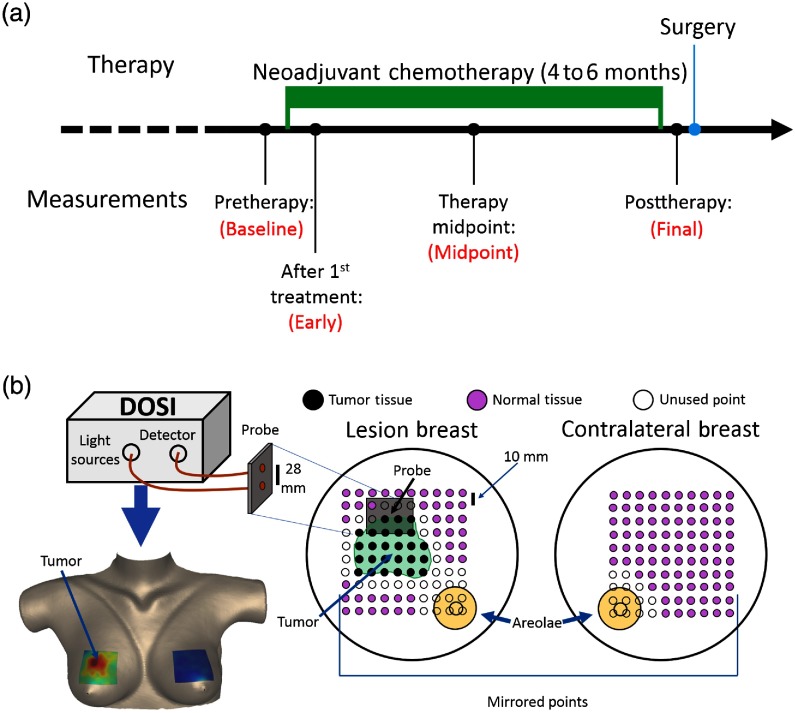
Timeline and schematic of DOSI measurement during NAC. (a) Each enrolled subject underwent NAC for a period of 4 to 6 months. DOSI measurements were made at four timepoints throughout the course of therapy: (1) baseline—prior to the administration of therapy, (2) early—5 to 10 days after the first dose of therapy, (3) midpoint—the midpoint of the therapy regimen, (4) final—at least 7 days after the final dose of therapy and prior to tumor resection. Note that some subjects are missing data at one or more of the nonbaseline timepoints, and the measurements at the final timepoint were not used due to their limited predictive utility. (b) Top left: DOSI instrument and probe. Right: a grid of points, over a surface area ranging from 7  cm×7  cm to 15  cm×16  cm, were measured on the lesion-bearing breast. This grid was chosen to encompass both the tumor and a portion of the surrounding healthy tissue. The grid of points was marked using a transparency, which was then used to mirror the grid for measurements on the contralateral breast. The transparency was also used to ensure consistent measurement locations across all timepoints. The tumor region was chosen to be all contiguous points with magnitude greater than half of the maximum TOI measurement. The tumor-bearing breast normal region was defined as all points outside the tumor region and areola, excluding a 1-cm margin around both the tumor and areola. The contralateral breast normal region was defined as all measured points, excluding the areola and a 1-cm margin around the areola. Bottom left: a sample DOSI image of the TOI contrast mapped onto a 3-D breast surface (see Sec. 2).

The DOSI instrument measured subjects using a handheld probe (handpiece) placed in contact with the patient’s breast. Four timepoints were acquired throughout the course of each patient’s NAC regimen^13^ (see Fig. 1). The first measurement (baseline) occurred prior to the first dose of chemotherapy. The second measurement, which is referred to as the early measurement timepoint, was performed between 5 and 10 days after the first chemotherapy treatment. The third measurement (midpoint) occurred in the middle of the therapy regimen, and a final measurement was made after the completion of therapy but prior to tumor resection. During each subject’s baseline measurement, a grid of ∼50 to 240 points that encompassed both the palpated tumor region and the surrounding normal tissue was measured on the lesion-bearing breast. A mirrored grid of points was measured on the contralateral breast (see Fig. 1). These measurement grids were recorded using a hand-marked transparency film that was produced for each subject in order to guide DOSI handpiece placement to the grid points during each measurement session as previously described.^13^

### 2.3 Statistical and Analytic Methods

In this study, we trained a logistic regression algorithm to discriminate between responders and nonresponders based on DOSI-measured parameters (list of parameters available in Table 2 in the Appendix). The tumor region for each subject was determined using a TOI $\left[\mathrm{TOI}=(\mathrm{HHb}\cdot H_{2}O)/\text{lipid}\right]$. This TOI parameter has been empirically shown to differentiate malignant tissue from normal tissue in the breast.^19^ The full-width-at-half-maximum contour around the point of maximum TOI in the baseline measurement of the lesion-bearing breast was designated as the edge of the tumor region. This region remained constant throughout all longitudinal measurements for a given subject. The normal region on the lesion-bearing breast was defined as all points outside the tumor region excluding the areola and 1-cm margins around the areola and tumor region (see Fig. 1). These margins were not included in the normal region to limit signal contamination due to the partial volume effect.

**Fig. 2:**
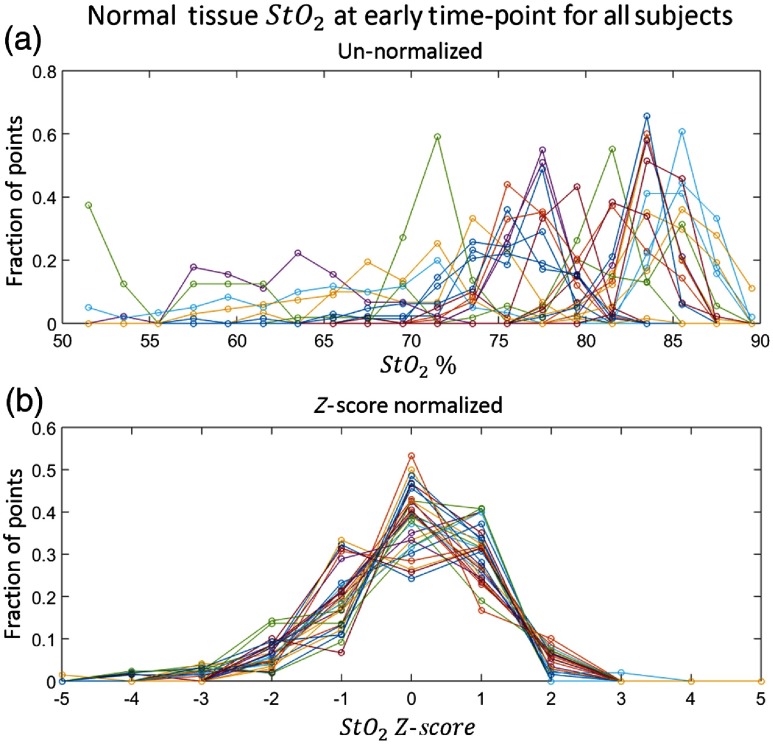
Histograms of the early timepoint normal tissue $S_{t}O_{2}$ for all subjects. (a) Fractional histograms of the unnormalized $S_{t}O_{2}$ of the normal tissue on the tumor-bearing breast at the early timepoint for each subject. Each line represents a different subject. (b) Fractional histograms of the z-score normalized log-transformed $S_{t}O_{2}$ data of the normal tissue on the tumor-bearing breast at the early timepoint for each subject. Each line represents a different subject. Note that with the z-score normalization, the distributions for all subjects have the same mean and an approximately Gaussian distribution. This effect is consistent across all measured parameters and timepoints.

In practice, significant inter- and intrasubject variation in optically measured physiological parameters of the breast can arise,^21^^,^^39^ and these systemic variations can bias the logistic regression. Moreover, the optically measured tissue parameters are not normally distributed (see Fig. 2).

To remedy these issues, we introduce and employ a z-score normalization method to define target variables for prediction of pathologic response. Briefly, the natural logarithm of each data point is first taken. Then, the mean and standard deviation of a normal (healthy) region of tissue are used to transform raw tumor data into z-score data as in

$$Z_{j}=\frac{\ln\;X_{j}-\left\langle \ln\;X_{j_{\text{Norm}}}\right\rangle }{\sigma [\ln\;X_{j_{\text{Norm}}}]}.$$ (1)

Here, $X_{j}$ is the unnormalized j’th measured parameter in the tumor region, $X_{j_{\text{Norm}}}$ is the unnormalized j’th measured parameter in the normal (healthy) region of the tumor-bearing breast, $\left\langle \ln\;X_{j_{\text{Norm}}}\right\rangle$ represents the mean over all points in the normal (healthy) region, and $\sigma [\ln\;X_{j_{\text{Norm}}}]$ represents the standard deviation over all points in the normal (healthy) region. $Z_{j}$ is then the tumor region z-score relative to the healthy tissue for the j’th parameter. Each $Z_{j}$ parameter was averaged over all spatial points in the tumor region for a given subject and timepoint. As a result, the logistic regression algorithms can utilize a single tumor quantity for each subject, for each timepoint, and for each measured parameter.

Thus every predictor data point used in the regression model is measured in units of standard deviations from the mean of a given parameter in healthy tissue. In addition to transforming all parameters to be approximately the same magnitude, this method better accounts for the intersubject systemic variations by finding the difference of each parameter from the mean value of the normal (healthy) tissue. It also more fully accounts for intrasubject variation in healthy tissue by normalizing with the healthy tissue standard deviation. A concrete example of this statistical transformation scheme is shown in Fig. 2 for early timepoint tissue oxygen saturation. In this study, we explored z-score normalization schemes that defined the normal region as either the healthy breast (excluding the areola) or all tissue on the lesion breast outside a certain margin of the tumor region (excluding the areola). See Fig. 1 for a graphical representation of these different normalization regions.

In the logistic regression framework, a response parameter for a given model is defined as

$$R^{i}=\beta_{o}+\overset{N_{j}}{\underset{j=1}{\sum }}\beta_{j}\cdot Z_{j}^{i}.$$ (2)

Here, $R^{i}$ is the given model’s log odds of response for the i’th subject, $\beta_{o}$ is the intercept term of the fitted weight vector, $\beta_{j}$ is the weighting term for the j’th measured parameter used in the model, $Z_{j}^{i}$ is the z-score for the j’th measured parameter of the i’th subject, and $N_{j}$ is the number of parameters used in a particular model. The full weight vector $\overset{\to }{\beta }$ is

$$\overset{\to }{\beta }=[\beta_{o},\beta_{1},\ldots ,\beta_{N_{j}}].$$ (3)

The $\vec{\beta }$ weight vector is fit using MATLAB’s native logistic regression function, mnrfit.^40^ The response parameter R can then be transformed into a probability of response parameter $P_{R}$ using a logistic function

$$P_{R}=\frac{1}{1+e^{-R}}.$$ (4)

The parameter $P_{R}$ represents the probability that a subject will achieve pCR. It has a range from 0 to 1, and it can readily be used to predict each subject’s status as either a pathologic complete responder, or noncomplete responder, depending on threshold levels.

**Fig. 3:**
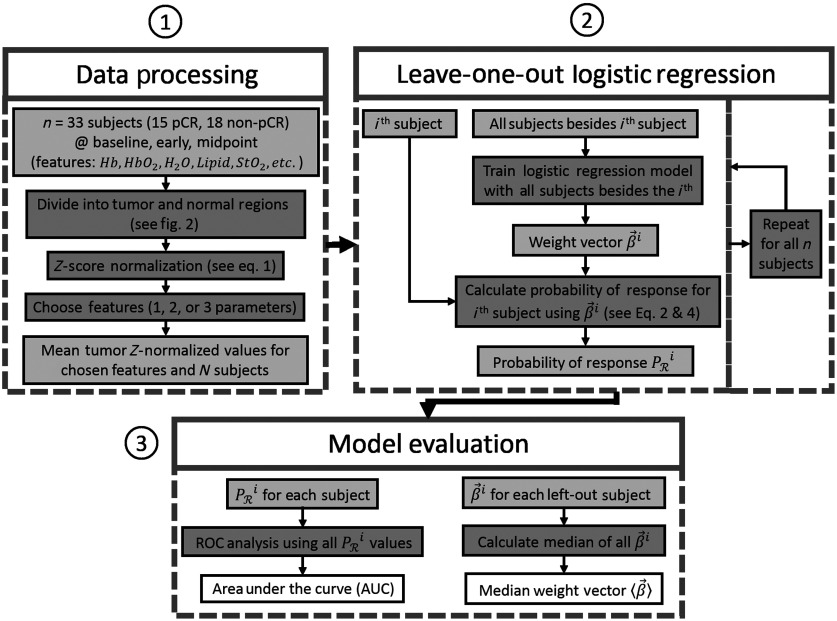
Data analysis flowchart. (1) Data processing—measured quantities at all spatial points and all n subjects across the first three timepoints are first divided into tumor and normal (healthy) regions (see Fig. 1). All tumor points are then z-score normalized to their respective normal (healthy) regions [see Eq. (1)], and the mean is taken for a given subject and timepoint. Finally, one-, two-, or three-model parameters are chosen from among the combinations of measured quantities and timepoints as model inputs. (2) Leave-one-out logistic regression—a set of n logistic regression algorithms are performed, each of which leaves out a single subject from the training data and produces a ${\vec{\beta }}^{i}$ weight vector. Each ${\vec{\beta }}^{i}$ is then used to calculate the probability of response for the subject left out of the given training set [see Eqs. (2) and (4)]. (3) Model evaluation—ROC analysis is performed using the calculated $P_{R}^{i}$ values to determine the AUC and a median weight vector $\left\langle \overset{\to }{\beta }\right\rangle$ is calculated from the n resulting ${\overset{\to }{\beta }}^{i}$ vectors.

Because we are working with a small dataset, we employed a leave-one-out validation protocol^41^ to test the regression model. Briefly, a series of logistic regression models are created for each parameter set we wish to test, and each of these models leaves one of the subjects out of the dataset (see Fig. 3). The weight vector created by each of these models ${\overset{\to }{\beta }}^{i}$ is the weight vector created when the i’th subject is left-out; it is used to produce a probability of response prediction for the i’th subject $\left(P_{R}^{i}\right)$, which is independent of the ${\overset{\to }{\beta }}^{i}$ model. This well-known approach provides the most robust and least biased validation given our sample size, which precludes the use of a significantly large independent test set.^41^ For completeness, we compared the leave-one-out protocol to other methods. For example, we also tested k-fold cross validation with k=3, 5, and 10; these schemes produced $\overset{\to }{\beta }$ vectors and AUC values that were similar to those of the leave-one-out protocol.

The quality of the resultant models was empirically determined using DeLong’s method for the area under the curve (AUC) and 95% confidence interval of a receiver-operating-characteristic (ROC) analysis graph for the $P_{R}$ parameter.^42^ The ROC analysis is performed using each of the individual leave-one-out models, and the reported weight vector $\left\langle \overset{\to }{\beta }\right\rangle$ will be the median $\vec{\beta }$ from the series of models created for each parameter set, with the interquartile range (IQR) of these models reported as uncertainty. We also calculated a single-logistic regression model run across the entire dataset; it produced very similar $\vec{\beta }$ vectors to the median $\vec{\beta }$ vector approach described above.

Models based on the z-scores of each single measured parameter (HHb, ${\mathrm{HbO}}_{2}$, lipid, $H_{2}O$, ${\mathrm{Hb}}_{T}$, $S_{t}O_{2}$, and TOI) at the baseline, early, and midpoint timepoints were tested, and the most predictive models were chosen using the AUC value as a criterion. To explore any additional benefit from multivariate models, combinations of two and three measured parameters were also evaluated. Models with more than three parameters were not tested to avoid overfitting. Data from the final timepoint were not used because our focus in this work is on early diagnosis.

Other, more commonly used, normalization methods were also tested to demonstrate the improvement in predictive ability provided by z-score normalization. These comparisons included tumor-to-normal ratio normalization without information about the normal tissue heterogeneity, as well as raw tumor physiological values without normalization, and baseline-normalized values, which represent changes in the measured parameters over the course of the therapy regimen. All statistical analysis was performed using MATLAB R2015a (The Mathworks, Inc., Natick, Massachusetts, USA).^40^

## 3 Results

The final data set was derived from n=33 subjects who had complete data sets at the baseline and midpoint timepoints. For models that used measured parameters from the early timepoint, slightly fewer subjects were used (n=29) due to missing data at this timepoint. All subjects had biopsy-confirmed invasive carcinomas and underwent an NAC regimen determined by their physicians.^13^

**Fig. 4:**
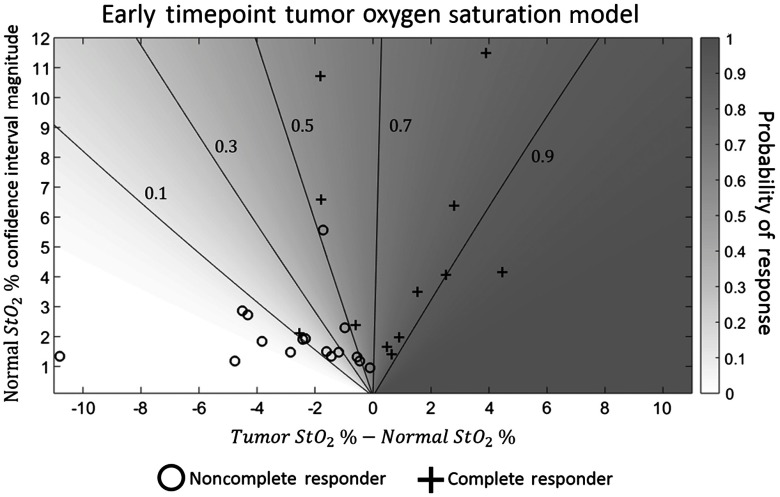
Tumor and normal $S_{t}O_{2}$ versus probability of response. This graph shows the probability of response predicted by the regression model using only early timepoint $S_{t}O_{2}$ (see Fig. 5). Contour lines of constant probability are also included. The probability of response (shading) is plotted versus the difference between the absolute tumor region percent oxygen saturation and the absolute normal region percent oxygen saturation (horizontal axis), and the size of the confidence interval for the absolute normal region oxygen saturation, corresponding to one standard deviation in the log-transformed data (vertical axis). Note that the oxygen saturation in this figure is not log-transformed or z-score normalized. Each cross represents a subject that was a pathologic complete responder while each circle indicates a nonresponding subject. All subjects that had tumor regions with absolute oxygen saturations that were higher than their normal regions achieved pCR. Subjects whose tumor regions were only slightly hypoxic relative to their normal regions were more likely to achieve pCR if the subjects’ normal regions had larger confidence intervals. These observations indicate that a subject is likely to be a pathologic complete responder if the oxygen saturation of the tumor region is either higher than that of the normal region or well within the normal region’s confidence interval. A subject whose tumor was significantly hypoxic relative to the normal tissue was likely to be a nonresponder.

**Fig. 5:**
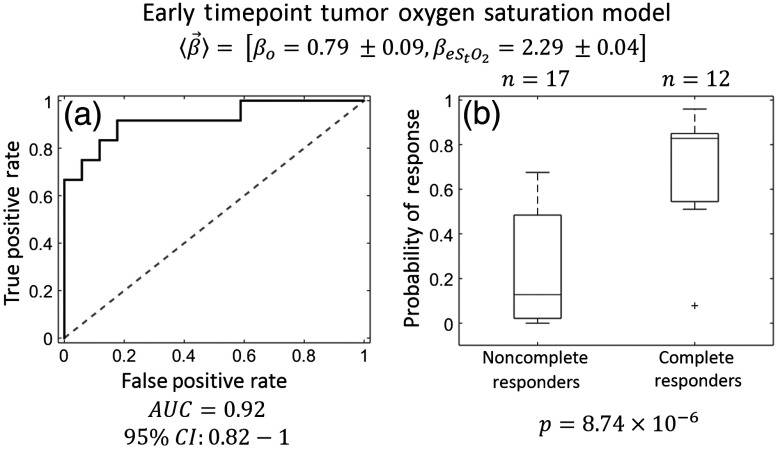
Early timepoint oxygen saturation prediction model. The model providing the best predictions used the early timepoint tissue oxygen saturation (${\mathrm{eS}}_{t}O_{2}$). The median weight vector $\left\langle \overset{\to }{\beta }\right\rangle =[\beta_{o}=0.79\pm 0.09,\beta_{{\mathrm{eS}}_{t}O_{2}}=2.29\pm 0.04]$ indicates that tumors that are not hypoxic relative to the normal tissue on the tumor breast are more likely to be pathologic complete responders to chemotherapy. (a) ROC analysis of ${\mathrm{eS}}_{t}O_{2}$ model—this model produced an AUC=0.92 (95% CI: 0.82 to 1), indicating excellent predictive value. (b) Boxplots of probability of response—the probability of response boxplots, divided into subjects that achieved pCR (n=12) and subjects that did not achieve pCR (n=17), indicate clear separation between the two groups using this model ($p=8.74\times 10^{-6}$ using a two-sided student’s t-test). The hinges of the boxplots represent the first and third quartiles of the data, the whiskers represent the range of measurements within a distance 1.5× the IQR, and the cross represents an outlier. Note that there is no overlap between the IQRs of the probability of response of the complete responders and noncomplete responders.

**Fig. 6:**
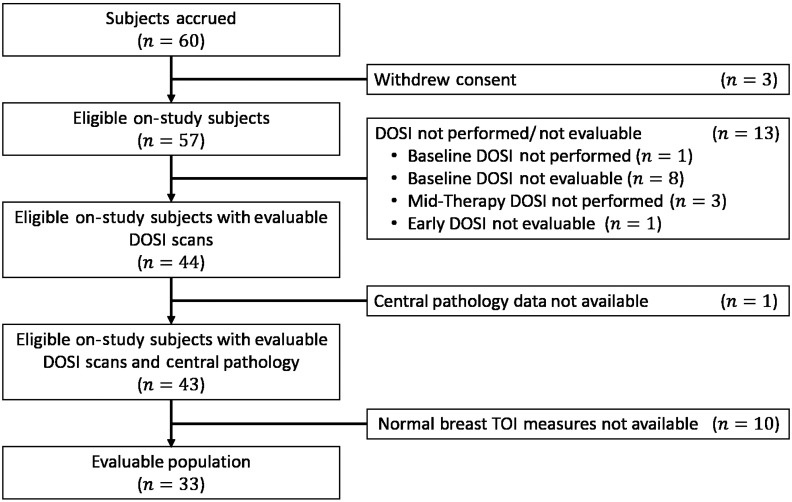
Subject exclusion chart. Of the 60 subjects accrued for this study, n=3 withdrew consent, n=1 did not have central pathology data, and n=10 were excluded for lack of normal tissue measurement. The other n=13 subjects were excluded due to lack of baseline DOSI measurement (n=1), baseline DOSI measurements that were not evaluable (n=8), lack of midpoint DOSI measurement (n=3), or too few normal region points were available (n=1). This subject population is identical to the population used in the initial ACRIN 6691 study^13^ except that one fewer subject was used. This additional excluded subject did have a normal tissue measurement but not a sufficient number of spatial points in the normal region to perform the necessary standard deviation calculation [see Fig. 2 and Eq. (1)].

For the logistic regression algorithm, z-score normalization to the healthy tissue on the lesion breast, as opposed to normalization to the contralateral breast, produced more predictive models. Recall that we derive z-score data for multiple data types (${\mathrm{HbO}}_{2}$, HHb, ${\mathrm{Hb}}_{T}$, $S_{t}O_{2}$, $H_{2}O$, and lipid) at multiple timepoints (baseline, early, and midpoint) (all data available in Table 3 in the Appendix). The single best regression model used only the early timepoint tissue oxygen saturation (${\mathrm{eS}}_{t}O_{2}$). The weight vector for this model was $\left\langle \overset{\to }{\beta }\rangle =[\beta_{o}=0.79\pm 0.09,\beta_{{\mathrm{eS}}_{t}O_{2}}=2.29\pm 0.04\right]$. This finding suggests that, at early timepoints, tumors that are not hypoxic relative to the surrounding normal tissue, or tumors that are only slightly hypoxic and within the normal region’s confidence interval, are more likely to be pathologic complete responders to NAC. By contrast, tumors that were significantly hypoxic relative to the normal tissue were likely to be nonresponders (see Fig. 4 for data summary in traditional units). When ROC analysis was performed, this model produced an AUC=0.92 with a 95% confidence interval of AUC=0.82 to 1 (see Fig. 5). Additionally, the small uncertainties of the $\left\langle \overset{\to }{\beta }\right\rangle$ components, relative to the median, indicate that the fitted $\left\langle \overset{\to }{\beta }\right\rangle$ did not vary significantly across the leave-one-out validation protocol.

Two- and three-parameter models did not improve upon the single-parameter model AUC. Higher-order models, e.g., four-parameter, were not considered in order to avoid overfitting of the data.

Notably, in addition to the early timepoint oxygen saturation, a two-parameter model using only baseline data provided an AUC=0.83 with a 95% confidence interval of AUC=0.70 to 0.97, thus enabling an even earlier prediction of a subject’s pCR status, albeit with lower accuracy than the early timepoint oxygen saturation. This two-parameter model incorporated the baseline oxygen saturation (${\mathrm{bS}}_{t}O_{2}$) and water concentration (${\mathrm{bH}}_{2}O$), and the median weight vector was $\left\langle \overset{\to }{\beta }\rangle =[\beta_{o}=0.14\pm 0.09,\beta_{{\mathrm{bS}}_{t}O_{2}}=1.69\pm 0.06,\beta_{{\mathrm{bH}}_{2}O}=0.65\pm 0.03\right]$. Again, the uncertainties in the $\left\langle \overset{\to }{\beta }\right\rangle$ components for $H_{2}O$ and $S_{t}O_{2}$ are small, signifying a consistent fitted model across the leave-one-out validation procedure. The fact that $\beta_{{\mathrm{bStO}}_{2}}>\beta_{{\mathrm{bH}}_{2}O}$ indicates that the oxygen saturation is a more significant predictor of pCR than water concentration at the baseline timepoint. As with the early timepoint model, subjects with hypoxic tumors were less likely to achieve pCR.

For comparison, additional models were produced that (1) used the contralateral breast for z-score normalization, (2) used tumor-to-normal ratio normalization, i.e., with no information about the standard deviation of the normal region, and (3) used no normalization. With contralateral z-score normalization, instead of z-score normalization to the healthy tissue on the tumor-bearing breast, the aforementioned early (${\mathrm{eS}}_{t}O_{2}$) and baseline (${\mathrm{bS}}_{t}O_{2}$ and ${\mathrm{bH}}_{2}O$) models had AUC values of 0.67 and 0.64, respectively. With simple tumor-to-normal ratio normalization, the same two-parameter sets produced AUC values of 0.80 and 0.67, and when completely unnormalized data were used, the ${\mathrm{eS}}_{t}O_{2}$ model produced an AUC=0.75 while the ${\mathrm{bS}}_{t}O_{2}$ and ${\mathrm{bH}}_{2}O$ model produced an AUC=0.68. Thus, for these parameter sets, z-score normalization to the healthy tissue in the tumor-bearing breast provided the best results.

## 4 Discussion

By application of a logistic regression model using z-score normalized DOSI measurements, we derived a robust predictor of response (AUC=0.92; 95% CI: 0.82 to 1) within the first 10 days after a subject’s initial chemotherapy dose. Using an optimally chosen cutoff value of $P_{R}=0.50$, which maximizes the sum of the sensitivity and specificity, this model provided an overall classification accuracy of 86% (25 of 29 subjects), including a positive predictive value of 79% for subjects predicted to achieve pCR (11 of 14), and a negative predictive value of 93% for subjects predicted to not achieve pCR (14 of 15). Prediction of response at this therapy timepoint was a secondary aim of the ACRIN 6691 protocol^13^ and could, with further validation, enable clinicians to modify the patient’s therapeutic plan after a single dose. This ability holds potential to improve patient outcomes and prevent unnecessary side effects from ineffective treatments.

The best model indicated that low $S_{t}O_{2}$ at the early timepoint relative to the surrounding normal tissue was predictive of nonresponse to chemotherapy. This observation suggests that tumors that are well-perfused in the early stages of treatment, and therefore are not hypoxic relative to healthy tissue, may receive chemotherapy more efficiently. Such tumors are also typically more responsive to therapy than hypoxic tumors, which often exhibit resistance to treatment.^43^^,^^44^ Additionally, the lack of hypoxia in complete responders could indicate a decreased oxygen demand due to suppression of tumor metabolism, a condition previously shown to be correlated with response to therapy.^45^

Additionally, the two-parameter model using only the baseline $S_{t}O_{2}$ and water concentration (AUC=0.83; 95% CI: 0.70 to 0.97) also indicated that higher $S_{t}O_{2}$ is correlated with pCR. Though the AUC value is lower for this model compared to the early timepoint $S_{t}O_{2}$ model, prediction of response prior to the initiation of therapy offers additional clinical utility. These models are also consistent with previous studies, which have observed correlation between pCR and optically measured tissue oxygen saturation prior to the start of therapy^31^ and after the first dose.^29^

Previous diffuse optical studies of response to breast cancer NAC have correlated temporal changes in measured physiological parameters with response to treatment.^24^^–^^30^^,^^32^ We compared our technique to this approach in the current study. However, even the most predictive of the models derived in this analysis that used the change in DOSI physiological parameters between the baseline and early timepoints only produced an AUC=0.63. The temporal change models of $S_{t}O_{2}$, in particular, could be limited by the large intersubject dispersion of the baseline oxygen saturation; this large dispersion prevents the change in $S_{t}O_{2}$ from the baseline to early timepoint from accurately reflecting the oxygenation state of the tumor relative to the normal region. By contrast, the model we have presented in this contribution does not depend on the baseline $S_{t}O_{2}$ and, as such, is not affected by intersubject baseline variation.

Z-score normalization was implemented to place all parameters on the same magnitude scale, which mitigates systemic physiological differences among the subject population and accounts for the systemic effects of chemotherapy. For comparison, we also investigated models that used fully unnormalized data and tumor-to-normal ratio normalization. However, since neither model incorporates healthy tissue standard deviation, neither model accounts for the heterogeneity of normal tissue. With tumor-to-normal ratio normalization, a one-parameter model with early timepoint $S_{t}O_{2}$ produced an AUC=0.80, and the two-parameter model with baseline timepoint $S_{t}O_{2}$ and $H_{2}O$ produced an AUC=0.67. The AUC values for the same models but with no normalization were even lower (AUC=0.75 and AUC=0.67, respectively). Thus z-score normalization improves the predictive power of the tissue oxygen saturation logistic regression models.

For completeness, several other models were explored that did not incorporate the baseline or early $S_{t}O_{2}$. Some of these produced predictions of response to therapy that were significant (AUC≈0.75 to 0.80). However, in addition to having lower AUC values, these other models relied on data from the midpoint timepoint, which increases the time-to-prediction of response by ∼2 months. Furthermore, the early timepoint measurements typically occur before significant anatomic changes in tumor size arise. This feature enables the DOSI measurement to sample known tumor tissue more easily; at the midpoint of therapy, by contrast, the tumor size has decreased and signal contamination between the malignant and healthy tissue can occur and limit the ability of DOSI to determine tumor physiological parameters accurately. Note also that the physiological predictions of these other models were consistent with our two primary prediction models.

Another interesting and potentially important finding of the present work is that the best models used z-score normalization to the normal tissue on the lesion breast rather than the contralateral breast. If, instead, the contralateral breast was used, our one-parameter early $S_{t}O_{2}$ model produced an AUC=0.67, and the two-parameter model with baseline $S_{t}O_{2}$ and $H_{2}O$ produced an AUC=0.64. The comparatively better quality of the tumor breast z-score normalized models suggests that measurement of the contralateral breast is less important for early prediction of response to therapy than previously thought. If this is true, then the paradigm could eliminate the need for contralateral measurement and reduce imaging time.

The results we have presented provide evidence for early prediction of response with AUC results that are comparable to other modalities, such as MRI,^46^^,^^47^ FDG-PET,^11^^,^^47^^,^^48^ and biomarker analysis.^49^^,^^50^ Some of these studies produced predictions prior to or within the first 10 days of treatment initiation,^48^^–^^50^ whereas other approaches relied on imaging that occurred either after 6 weeks of NAC,^46^ at the midpoint of therapy,^11^ or after the completion of NAC.^47^ The potential advantage of the logistic regression DOSI model is premised on its unique combination of accurate prediction at an early timepoint in therapy and its portability, low cost, and lack of ionizing radiation.

The primary limitations of this study are the relatively small number of subjects and the highly variable chemotherapy regimens across the subject population. Additionally, the initial study had a fairly high dropout rate,^13^ introducing a potential bias into the statistical analysis. The dropout rate is likely to be artificially elevated in this study due to the difficulties inherent in translating an experimental imaging technique into a multisite setting for the first time.^13^ We do not anticipate that these issues will affect the DOSI technique moving forward. Finally, although the initial ACRIN 6691 trial was a prospective study, this z-score parameter imaging metric was retrospectively optimized using a standard leave-one-out protocol for multiple potential models. The leave-one-out technique limits overfitting and enhances the generalizability of the prediction metric;^41^ it has been extensively used by the cancer community.^31^^,^^46^^,^^51^^–^^53^ Of course, a fully prospective validation of this single prediction model, as opposed to the series of models tested here, will be necessary prior to clinical adoption.

Per the first limitation noted above, application of this model to a prospective study with a larger subject population is a natural course of action. Importantly, because the DOSI instrumentation has been shown to provide consistent performance over time, across multiple instruments, and across multiple measurement sites,^38^ we anticipate that the weight vector derived for the early timepoint $S_{t}O_{2}$ (see Fig. 5) could be used with z-score normalized measurements in future DOSI studies to calculate a probability of response, i.e., without creating a logistic regression model for each population. In this case, the future study would serve as a direct, independent test set for the results obtained by our current model. Additionally, a logistic regression could also be run on this larger data set to derive an improved prediction model based on a larger training set. If a future study was performed on a significantly different patient population, e.g., patients with tumors in nonbreast tissue, then deriving a weight vector via logistic regression would likely be beneficial.

In addition to providing evidence to further corroborate the results of this pilot investigation, the larger subject population may enable stratification of the subject population by tumor subtype and/or chemotherapy regimen. Our current results are reported for a diverse patient population, various tumor molecular subtypes, and an assortment of chemotherapy regimens (see Table 1). Tumor subtypes may have different levels of tissue oxygen saturation and may respond to chemotherapy differently.^3^^,^^54^ The physiological mechanisms of chemotherapy regimens also vary. Thus, especially for parameters at the early timepoint, response prediction might be improved by creating individual models for different classes of chemotherapy and/or different tumor subtypes. Also, independent hypoxia biomarkers, such as carbonic anhydrase IX, and measurements of vascular density, such as CD31 staining or DCE-MRI, can be collected at similar timepoints and may enable better understanding of the mechanisms responsible for correlations between tissue oxygen saturation and response. Exploration of these questions should be possible in a larger study.

## 5 Conclusion

Logistic regression modeling of z-score normalized physiological parameters measured by DOSI was presented and found to predict pCR to NAC. The best model successfully predicted pCR (AUC=0.92; 95% CI: 0.82 to 1) using tumor and normal tissue oxygen saturation measured within the first 10 days after the initial dose of therapy based on data from the ACRIN 6691 clinical trial.^13^ This model suggests that if tumors are hypoxic relative to the surrounding normal tissue, then they are less likely to achieve pCR. These early predictions of therapeutic efficacy are based on quantitative DOSI measurements of tumor (and normal) tissue functional parameters, rather than changes in tumor size, and the z-score normalization of the tumor physiological data yielded improved prediction models compared to tumor-to-normal ratio or unnormalized data. Prospective validation is still needed to confirm these promising results. With this validation, DOSI and logistic regression methods could be used early in NAC to optimize treatment outcomes for individual patients.

## Appendix

This appendix contains details about the subject exclusion criteria (Fig. 6) and a more complete accounting of measured z-score parameters for responders and nonresponders at all timepoints (Tables 2 and 3).

**Table 2:** Definitions of measured DOSI parameters and their methods of calculation. HHb, ${\mathrm{HbO}}_{2}$, lipid, and $H_{2}O$ concentration are all fit directly using the measured intensities throughout the wavelength range. ${\mathrm{Hb}}_{T}$, ${\mathrm{StO}}_{2}$, and TOI are all derived from the fit parameters.

Parameter	Meaning	Calculation Method
HHb	Deoxy-hemoglobin concentration	Multispectral fit of absorption
${\mathrm{HbO}}_{2}$	Oxy-hemoglobin concentration	Multispectral fit of absorption
Lipid	Lipid concentration	Multispectral fit of absorption
$H_{2}O$	Water concentration	Multispectral fit of absorption
${\mathrm{Hb}}_{T}$	Total hemoglobin concentration	${\mathrm{Hb}}_{T}=\mathrm{HHb}+{\mathrm{HbO}}_{2}$
$S_{t}O_{2}$	Tissue oxygen saturation	$S_{t}O_{2}={\mathrm{HbO}}_{2}/{\mathrm{Hb}}_{T}$
TOI	Tissue optical index	$\mathrm{TOI}=(\mathrm{HHb}\cdot H_{2}O)/\text{lipid}$

**Table 3:** Median z-score values with IQRs for each measured parameter and timepoint, separated by pCR status.

	Baseline median z-score (IQR)		Early median z-score (IQR)		Midpoint median z-score (IQR)	
	pCR	Non-pCR	pcR	Non-pCR	pcR	Non-pCR
${\mathrm{HbO}}_{2}$	1.5 (1.1, 1.8)	1.0 (0.4, 1.9)	1.4 (0.8, 1.7)	0.6 (0.1, 1.1)	0.6 (0.4, 1.1)	0.5 (0.2, 1.3)
HHb	2.2 (1.5, 2.7)	1.9 (1.5, 4.0)	1.5 (1.0, 1.9)	1.7 (1.2, 3.4)	1.2 (0.9, 1.6)	1.4 (0.9, 3.0)
${\mathrm{Hb}}_{T}$	1.8 (1.5, 2.4)	1.4 (0.8, 2.2)	1.4 (0.8, 1.8)	0.9 (0.5, 1.3)	1.0 (0.7, 1.4)	0.9 (0.5, 1.6)
$S_{t}O_{2}$	−0.2(−0.8,0.1)	−1.8(−2.3,−0.8)	0.4 (−0.3,0.5)	−1.2(−1.7,−0.4)	−0.4(−1.0,0.2)	−0.9(−1.8,−0.6)
$H_{2}O$	2.1 (1.5, 2.8)	1.8 (1.2, 3.1)	1.5 (1.3, 2.3)	1.2 (0.8, 3.2)	0.9 (0.4, 2.0)	1.3 (0.9, 2.3)
Lipid	−1.8(−2.9,−1.4)	−1.4(−2.5,−0.1)	−1.0(−1.8,−0.6)	−0.9(−2.2,−0.1)	−0.7(−1.1,−0.1)	−0.9(−1.6,−0.6)
TOI	3.0 (1.7, 3.8)	2.4 (1.6, 4.1)	1.7 (1.2, 2.4)	1.5 (1.2, 4.0)	1.5 (0.9, 2.0)	1.5 (1.2, 3.2)
